# An Expanded Multi-scale Monte Carlo Simulation Method for Personalized Radiobiological Effect Estimation in Radiotherapy: a feasibility study

**DOI:** 10.1038/srep45019

**Published:** 2017-03-21

**Authors:** Ying Zhang, Yuanming Feng, Wei Wang, Chengwen Yang, Ping Wang

**Affiliations:** 1 Department of Biomedical Engineering, Tianjin University, Tianjin 300072, China; 2 Department of Radiation Oncology, Tianjin Medical University Cancer Institute and Hospital, Tianjin 300060, China; 3 East Carolina University, Greenville, NC 27834, USA

## Abstract

A novel and versatile “bottom-up” approach is developed to estimate the radiobiological effect of clinic radiotherapy. The model consists of multi-scale Monte Carlo simulations from organ to cell levels. At cellular level, accumulated damages are computed using a spectrum-based accumulation algorithm and predefined cellular damage database. The damage repair mechanism is modeled by an expanded reaction-rate two-lesion kinetic model, which were calibrated through replicating a radiobiological experiment. Multi-scale modeling is then performed on a lung cancer patient under conventional fractionated irradiation. The cell killing effects of two representative voxels (isocenter and peripheral voxel of the tumor) are computed and compared. At microscopic level, the nucleus dose and damage yields vary among all nucleuses within the voxels. Slightly larger percentage of cDSB yield is observed for the peripheral voxel (55.0%) compared to the isocenter one (52.5%). For isocenter voxel, survival fraction increase monotonically at reduced oxygen environment. Under an extreme anoxic condition (0.001%), survival fraction is calculated to be 80% and the hypoxia reduction factor reaches a maximum value of 2.24. In conclusion, with biological-related variations, the proposed multi-scale approach is more versatile than the existing approaches for evaluating personalized radiobiological effects in radiotherapy.

In the past two decades, the advances in modern external photon beam radiotherapy techniques have allowed us to sculpt steep dose gradients around the tumor, shrink the safety margins and reduce the normal tissue doses^1^. However, radiotherapeutic physics has arguably reached a boundary where further significant gains require an interdisciplinary effort of connecting physics and the underlying biology^2^. Until recently, biological response was generally estimated by macro physical quantities, i.e., dose and dose-volume (DV) parameters, or related biological models such as the biologically equivalent dose (BED)^3^ and equivalent uniform dose (EUD)^4^. In this way, the details of the specific biological mechanisms of radiation effect were covered by nonspecific (statistically averaged) parameters over relatively wide time and spatial scales so that the inherent random fluctuations of Ionizing radiations as well as the radiobiological effects are negligible. Moreover, the increase in relative biological effectiveness (RBE) for low energy X-rays has been observed experimentally for a range of biological endpoints^5^,^6^. It is noted that the actual value of the RBE can vary in different parts of a patient or beam by an amount that is much larger than the tolerance for clinical treatment planning^7^,^8^. Therefore, it is almost imperative to find a reliable and practical approach to quantitatively evaluate tissue response to radiotherapy in a more elaborate manner.

Current biological models, including the commonly used linear-quadratic (LQ) model, are mostly “top-down” models^9^. Specifically, complex biological systems are treated as a black box where physical quantity (e.g. absorbed dose) and biological quantity (e.g. the cell survival fraction) are correlated by an empirical fitting and the inherent unpredictable nature of biological systems is ignored. The idea of “bottom-up” radiobiological estimation approach has been proposed by Wang *et al*.^9^. By contract, the “bottom-up” approach aims to take into account of all the detailed mechanisms of the radiation interaction, through which one can understand how the radiation action take place and what the true difference among different therapies is. An ideal but sketchy program structure for developing a new multi-scale radiobiological model was initially proposed in ref. 9. In their purely theoretical structure, the program is based on the stochastic Monte Carlo (MC) procedures. During estimation procedure, various biological pathways are included such as the DSB yields, damage evolution and intercellular communication, etc. The application of MC techniques for radiotherapy outcome prediction was also highlighted by Naqa *et al*.^10^. In their views, the multi-scale approach would encompass the multi-scale parts of the tumorigenesis (atomic, molecular, tissue, organ) and various multi-scaled radiation-induced response stages (i.e. physical, chemical and biological) over the spatial and temporal axes.

Monte Carlo simulation is a useful approach to probe the underlying basis for the radiation effects. The track-structure technique in MC provides a way to determine the number and spatial configuration of damage clusters at molecular and sub-cellular levels^11^,^12^. However, the step-by-step tracking algorithm is extremely time consuming and thus have limited applications in higher dimensions^11^,^12^,^13^. To overcome this disadvantage, a fast and quasi-phenomenological Monte Carlo damage simulation (MCDS) algorithm was developed by Semenenko *et al*.^14^,^15^ to estimate the radiation-induced damages at cellular level. Similar to the threshold and probability models used in track structure simulation, MCDS used four adjustable parameters and the optimal parameter values were determined from an empirical fit to the results from track structure simulations and radiobiological experiments. It has been illustrated that default MCDS parameter values could adequately reproduce cluster yields for a wide range of particle types, kinetic energies and oxygen concentrations^16^.

There is comprehensive evidence that DNA double-strand breaks (DSBs) are a genotoxic form of DNA damage and closely related to higher order biological endpoint, i.e. cell death^17^,^18^. DSBs have been employed in many radiobiological investigations as indicator of the relative biological effectiveness (RBE)^19^,^20^. As organism cells have multiple damage repair mechanisms to ensure genomic integrity, it is important to quantify the effect in the radiobiological modeling. Over the past few years, many theoretical models have been studied, from quasi-empirical models, such as the widely used linear-quadratic (LQ) formula^21^, to more mechanistic kinetic models, such as the lethal–potentially lethal (LPL) model^22^ and the repair–mis-repair (RMR) model^23^. More recently, Stewart *et al*. proposed a two-lesion kinetic (TLK) model^24^ featuring a direct link between biochemical processing of the DSBs and cell killing. One advantage of TLK model is that it provides a satisfactory formalism in correlating the process of DSBs with cell killing^24^.

In this study, our goal was to develop a versatile approach to estimate the radiobiological effect of clinic radiotherapy using expanded multi-scale Monte Carlo simulation. A systematic model was created, which includes several sub-models and multi-scale MC simulations from macroscopic organ to microscopic cell levels using Geant4 MC Toolkit (as illustrated in Fig. 1). To calibrate the TLK model, multi-scale simulation was first conducted to replicate a radiobiological experiment and obtain optimal model parameters. Multi-scale modeling was then performed on a 3D radiotherapy plan of a lung cancer patient. The cell survival fraction (SF) of two representative voxels (isocenter and peripheral voxel of the tumor) were finally estimated and compared with those from LQ model. To demonstrate the capabilities of the model, the dependence of oxygen concentration on tumor cell killing was quantitatively analyzed.

## Material and Methods

In this study, all methods were carried out in accordance with relevant guidelines and regulations.

### Cellular damage database

In this study, MCDS program was used to generate the cellular DSB damage database. The default parameters in MCDS model were chosen as in the literature^16^. A series of simulations were conducted to generate the cellular DSB damage database with monoenergetic electrons from 50 eV to 6 MeV under various oxygen concentrations (0 to 100%). For each kinetic energy, 10000 nucleus with a diameter of 10 μm were simulated to ensure the mean standard error is less than 0.2%. The recorded yields of simple DSB (sDSB, two SSBs occur on opposite strands within 10 base pairs) and complex DSB (cDSB, an sDSB accompanied by at least one additional strand break within 10 base pairs) correspond to two DSB repair mechanisms - fast and slow DSB rejoining kinetics^25^,^26^, respectively.

### Damage accumulation algorithm

A spectrum-based accumulation algorithm^6^,^16^ was employed to compute the accumulated DSB damages for each nucleus. At micrometer cellular level, only electrons were considered to induce DNA strand break damages. The yield of each DSB damage can be computed by integrating microscopic dose within the nucleus and initial yield per unit dose from database over all kinetic energies of recorded electron spectrums. According to ICRU Report 36^27^, the microscopic absorbed dose is equal to the product of the number of particles passing through the nucleus times the microdosimetric quantity, i.e. frequency-mean specific energy. Therefore, the accumulated sDSB and cDSB yield of each nucleus can be computed as:


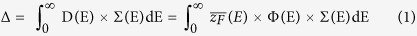


In Equation (1) is the total damage yield for sDSB or cDSB. D is the microscopic deposited dose, and Σ is the damage yield per Cell per Gy from predefined database. Φ represents the number of electrons that traverse the nucleus. 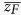 is the frequency-mean specific energy, which is calculated as:


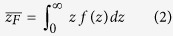


In Equation (2), specific energy z is defined as the quotient of energy imparted to a “microscopic” volume by a single event and the volume mass^28^. On a microscopic scale, the transferred energy is a stochastic quantity, so z is also a stochastic variable with a probability density function of f(z). A series of MC simulations were conducted using Geant4 toolkit to obtain the specific energy distributions of various monoenergetic electrons (E) that travels through a spherical nucleus of 10 μm in diameter. The electrons were uniformly distributed on the surface of the nucleus (s-randomness) with uniform angular distributions inwards. The secondary electron production threshold was set to be 250 eV using Penelope physical package. A total number of 10^6^ particles were used for each simulation. Total energy deposition of each primary particle was recorded and 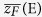 were thus computed and interpolated linearly to make a continuous curve in the range of 50 eV and 6 MeV for specific tissues. Estimation of DSB damage spectrums for each cell requires accurate particle spectrum and fluence into each nucleus, which was estimated using Geant4 multi-scale Monte Carlo simulation toolkit.

### Cell survival estimation model

#### TLK model algorithm

In TLK model, all DSBs are subdivided into simple and complex DSB. Each DSB damage has its own unique repair characteristics. Break-ends associated with both DSBs are allowed to interact in not only a linear mechanism but also a pairwise manner to form irreversible lethal and non-lethal chromosome aberrations. The dynamic of DSBs for each nucleus can be modeled by two nonlinear differential equations^29^


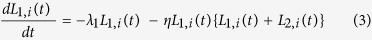



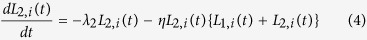


here *L*_1,i_ (t) and *L*_2,i_(t) are the numbers of sDSBs and cDSB, respectively, for i-th nucleus at time t. λ_1_ and λ_2_ are the repair probabilities of sDSBs and cDSBs by first-order linear repair mechanisms. Both λ’s can be related to the repair half time (denoted as τ_i_) of each kind of damages using λ_i_ = ln(2)/τ_i_. Parameter η is the second-order pair-wise interaction probability (h^−1^).

The time-dependent rate at which DSBs are converted into lethal damage is modeled by


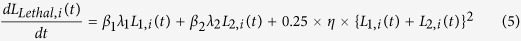


where β_1_ and β_2_ represent the fidelity of linear mis-repair mechanism for sDSBs and cDSBs respectively. The constant 0.25 indicates the possibility of forming a lethal chromosome aberration (i.e. dicentric) through pairwise damage interaction. According to previous investigations^29^,^30^, the survival probability for a cell with lethal damage of *L*_Lethal,i_ at given time T after irradiation is calculated as:


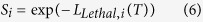


An in-house MATLAB software tool was developed for the modeling and calculations. Dynamic of the number of lethal damages in each cell and the survival probability were calculated at different time points thereafter. A random number was generated to determine whether the cell survives. This process was repeated for all nucleus models and the survival fraction was calculated as the biological endpoint.

#### TLK model calibration

Since the mechanism of cellular actions operates in approximately the same manner *in vitro* as *in vivo*, the parameters identified *in vitro* can be used for *in vivo* system^24^,^29^. Multi-scale MC method was utilized to mimic a radiobiological experiment. After that optimal TLK model parameters were obtained to best fit the experimental results. The parameters can be regarded as characteristic parameters for DNA damage repairing.

The cell survival data published by Bromley *et al*.^31^ was used for TLK model calibration. In this experiment, A549 cells (human non-small cell lung cancer cell line) cultured in a six-well plate were irradiated using a 6 megavoltage (MV) x-ray beam produced by a clinical accelerator (Varian Medical System, Palo Alto, California, USA). The center of the plate was aligned with the central axis of the treatment machine and the cell layer was positioned at the depth of 1.5 cm (i.e. the depth of maximum dose of the 6 MV beam). The plate was surrounded by Perspex to ensure full scatter conditions. A half beam radiation field was created using orthogonal jaws. After irradiation, the six-well plates were incubated and then fixed and stained with crystal violet. The colonies having more than 50 cells were manually counted as representing a surviving cell. Six replicate experiments were used to increase the accuracy of the cell survival estimation.

**Figure 2:**
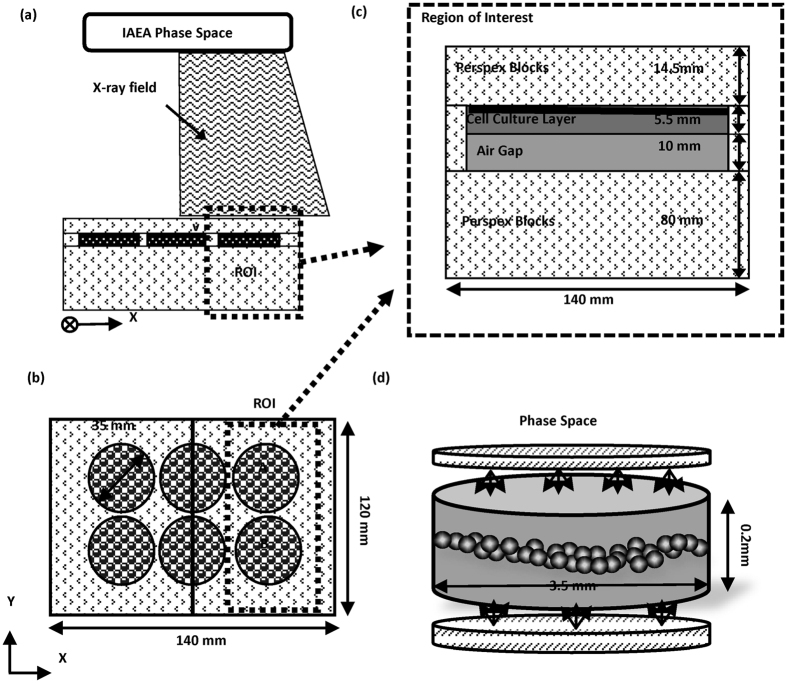
Diagram of geometries for TLK model calibration. (**a**) Whole geometry for first MC simulation. (**b**) Six-well plate at the cellular level. (**c**) Longitudinal section of the ROI region. (**d**) Geometry for secondary MC simulation.

In this study, two uniformly irradiated wells were chosen as Region of Interest (ROI) for TLK model calibration. The geometry of the first MC simulation strictly followed the experimental configurations, which are illustrated in Fig. 2(a–c). A phase space file of Varian Linac provided by the International Atomic Energy Agency (IAEA)^32^ was used as particle source. The Penelope physical package was used and the secondary electron production threshold was set to be 15 keV for the first simulation. The incident electron information (kinetic energy, position, and direction) of a cylinder cell layer of 0.2 mm in thickness was recorded and used for further simulation. As the probability of incident electrons scattered from the side surface of or created inside the thin cylinder volume is very low, they were neglected for simplification. And with our settings, incident photons were not considered either during the second MC simulation since most of the step-length between two photon interaction points is much larger than 0.2mm.

During the secondary MC simulation, two circular planar sources were created to represent the forward and backscattered electrons from top or bottom surface of the cylinder volume using the recorded energy and angular distribution information. A total number of 3000 spherical nuclei of 10 μm in diameter were randomly distributed in the middle level of the cell layer cylinder with a ±10 μm fluctuation along Z direction (Fig. 2(d)). To avoid artifacts due to lack of side scatter near edges, the whole cylindrical phantom was located in the center of a water box with a side length of 5 cm. To ensure accuracy of the incident spectrum, a 10 μm “Watch Volume (WV)^8^” ring was added out of each nucleus model to take most of the secondary electrons lower than 15 keV into consideration. Within the WV region, Geant4-DNA physical processes were activated once the energy of the electron was lower than 50 keV, otherwise Penelope physical processes were employed. The secondary electron production threshold was lowered down to 50 eV in this volume. And for the rest of the volume, only Penelope physical package was employed and secondary electron production threshold was set to be 15 keV. During the simulation, incident electron spectrum was recorded for each nucleus to perform the spectrum-based damage estimation. The DNA damage yield of each nucleus was first calculated and then converted linearly to corresponding macroscopic doses (0.1, 1, 2, 3, 4, and 5 Gy) for cell survival fraction estimation. To account the inherent diversity among cells, fast and slow half repair time were assigned by randomly allocating values from Gaussian distributions (μ = 0.25 or 8 h, σ = 0.1 or 1) to each nucleus^33^. The survival fraction was calculated by literately solving the differential equations through a repair time of 96 hours with a small time step of 0.001 h.

The objective function (OBJ) (as shown in Equation 7) was calculated between experimental and simulated cell survival data points to get the optimal model parameters:


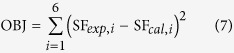


Parameter sensitivity analysis was first performed for the rest three parameters (i.e. β_1_, β_2_ and η) through changing one parameter within a plausible range of value while keeping other parameters constant. The one that altered OBJ the most was identified as the most sensitive parameter and was adjusted first. The optimal parameter values were obtained by minimizing OBJ to best fit the experimental results.

### Radiotherapy outcome estimation

To conduct multi-scale Monte Carlo simulation, the CT images of a lung patient were chosen with the approval of the Ethics Committee of Tianjin University Biomedical Engineering and the patient’s written consent. A 3D-CRT treatment plan was generated by an experienced physicist using the Pinnacle^3^ treatment planning system (TPS) (Philips Radiation Oncology Systems, Milpitas, California, USA). The prescription dose to PTV was 50 Gy in 25 fractions.

For the general MC simulation, patient geometry was built through resampling the CT images by a factor of 2. The voxel size was 2.54*2.54*2.5 mm^3^. The voxel grey values, representing CT numbers, were first converted to a given density and then to a material type. Different materials were associated with density ranges as specified in ICRU report No. 46^34^. Currently, the following ten materials were used to simulate patient tissues: air, lung (inhale), lung (exhale), adipose tissue, breast, soft tissue, muscle, liver, trabecular bone and dense bone. G4PhantomParametrization class of Geant4 was used to define voxel dimension, transformation, density and materials. Such parameterization was later used to directly locate phantom voxels crossed by the tracks. The option to skip boundaries between voxels with the same materials has been used and a correction algorithm was used to properly distribute the energy/dose in each voxel along the track^35^. The file of DICOM-RT structure set was used to define the body, target and organ at risk (OAR) regions. Secondary electron production threshold was set to be 1 mm in length within the body. The length cuts were internally converted to energy cuts according to particle type and materials.

The IAEA phase space file^32^ was adopted as the particle source for the general Monte Carlo simulation. Two pairs of Jaws and MLCs were also added in the accelerator box. The MLC leaves were built in the form of slice boxes with the exact positions for each leaf extracted from the leaf sequence files of the treatment plan. The phase space, jaws and MLCs could be rotated about isocenter as a whole. During the first MC simulation, the history numbers were set to 1*10^9^ for each beam to keep dose deposition uncertainty lower than 0.5% for most of the PTV voxels. The radiation patterns (particle type, track kinetic energy, position, and direction) of the isocenter voxel and a peripheral tumor voxel were saved for further simulation.

Before secondary MC, to deal with the heavy time consuming issue caused by the geometrical boundaries during the Geant4 particle tracking, an extra intermediate step was conducted. Homogeneous voxel model without nucleus was used for this simulation. Two superficial particle sources were created to represent incident photon and electrons respectively using previous recorded radiation patterns for each voxel through General Particle Source (GPS) in Geant4. The Penelope physical package was employed and the secondary electron production threshold was set to be 250 eV. The basic information of all generated secondary electrons within the voxel was saved into a phase space file for further simulation, and the electrons were then terminated.

During the secondary MC, a total of 10^5^ spherical nucleus models (10 μm in diameter) were randomly distributed in the voxel box with corresponding material and density. Several variance reduction techniques including electron range/region rejection, and electron track repeating^36^ were implemented to increase the efficiency. The simulation was monitored by calculating the mean LET of the incident electrons for 100 randomly chosen nucleus and terminated after the “stable status”, i.e. constant mean LETs for the chosen nucleus was achieved. As described above, the spectrum-based algorithm was employed to calculate the nucleus damage distribution. Damage patterns were linearly converted to the corresponding macroscopic dose obtained from the first MC simulation. The survival fraction of the voxels (SF_MC) were calculated by literately solving the differential equations of TLK model through a total repair time of 24 hours with sufficiently small time step of 0.005 h. The obtained SFs were then compared with those from the conventional LQ modeling (α = 0.2432 and β = 0.0257^31^). Moreover, cell survival fractions of the isocenter voxel were computed and compared under different oxygen concentrations. Hypoxia reduction factors (HRFs)^37^, i.e. ratio of dose required to produce the same cell killing effect as the normoxic condition, were calculated to quantify the oxygen effects.

## Results

### Cellular damage database

**Figure 3:**
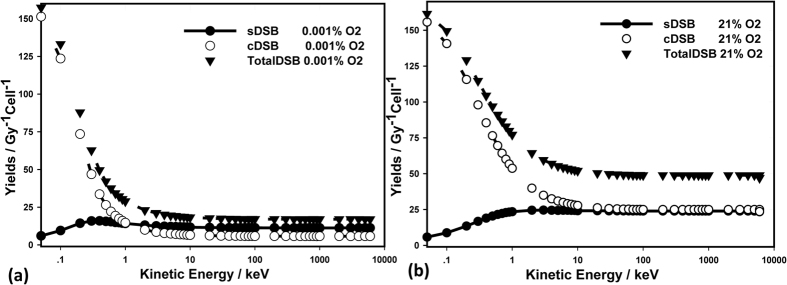
Mean DSB yields per Cell per Gy for (**a**) anoxic condition (0% Oxygen) and (**b**) normoxic condition (21% Oxygen).

MCDS package (Version 3.10 A)^14^ was used to generate the cellular damage databases. Figure 3 shows plots of the mean number of DSB per Gy per Cell for monoenergetic electrons under anoxic condition (0.001%) and normoxic condition (21%). For normoxic condition, total DSB yield is nearly constant at the energy level above 100 keV, whereas below 100 keV, it increases dramatically as incident energy decreases. This result suggests that different radiobiological effects may be due to the incident electron spectrum, or more specifically, the percentage of low energy electrons (i.e. <100 keV).

### TLK model calibration

**Figure 4:**
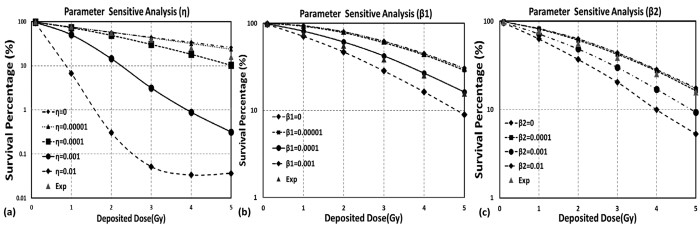
Parameter sensitivity analysis of TLK model. (**a**) Pair-wise interaction Probability, η. (**b**) probability of lethal mis-repair of sDSB, β_1_. (**c**) Probability of lethal mis-repair of cDSB, β_2_.

Accumulated nucleus dose were computed using the frequency-mean specific energy The mean nucleus dose was slightly lower than macroscopic deposited dose (i.e. 0.89 ± 0.055 Gy for 1 Gy). The distribution pattern of nucleus dose indicates that macroscopic value is not sufficient to represent the microscopic stochastic values. Figure 4 shows the results of parameter sensitive analysis. It is apparent that pair-wise interaction probability (η) has a significant impact upon cell survival results. This justifies the strategy of optimizing pair-wise interaction probability first. Globally optimized parameter values for the A549 cells were chosen as: β_1_ = 0.00026, β_2_ = 0.011, and η = 1.6E-05 (h^−1^). The obtained cell survival fractions are in good agreement with experimental data.

### Radiotherapy outcome estimation for patient

**Figure 5:**
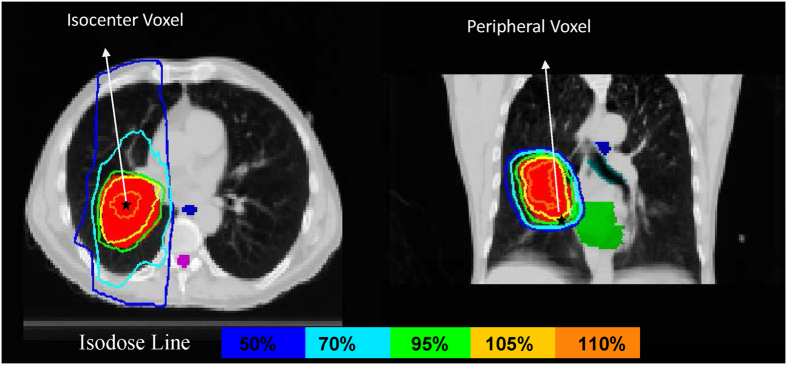
Isodose distribution of the lung cancer patient. The location of two chosen voxels is identified using star marks.

In the first MC simulation of a lung patient, final dose distribution was normalized so that 100% isodose surface encompassed 95% of PTV volume. The isodose distribution was shown in Fig. 5 with the location of two voxels identified. The macroscopic and microscopic characteristics of two chosen voxels under normoxic condition (21%) are summarized in Table 1. The deposited dose of the peripheral voxel is slightly lower than that of the isocenter voxel, but both are higher than the prescribed dose of 2 Gy per fraction. In addition, the peripheral voxel receives a slightly “left shifted” irradiation with smaller mean kinetic energy compared to the isocenter voxel.

At microscopic level, the MC simulations for each voxels were repeated three times and mean values of each indices were listed in Table 1. The nucleus dose and damage yields vary among all nucleuses within the voxel. The nucleus doses are 2.07 ± 0.5 Gy and 1.97 ± 0.52 Gy for the isocenter and peripheral voxels, respectively. Both are slightly lower than the macroscopic dose of the whole voxels. Total DSB yields are 111.5 ± 24.1 and 109.8 ± 25.3 respectively. Slightly larger percentage of cDSB yield is observed for the peripheral voxel (55.0%) as compared to the isocenter one (52.5%). The estimated SFs for the central and peripheral voxel are 55.8% and 57.9% with a standard deviation of 0.5% and 0.4%, respectively. The uncertainty in SF estimation is less than 0.9%, which has demonstrated the stability of the systematic model. Compared with the widely used LQ model, same trend in the relationship between macro-dose and radiation-induced cell death is observed in this study. However, the SF results are about 6.6% higher for both voxels, i.e. 55.1% vs 51.7% and 57.9% vs 54.3%, respectively. The difference between SFs of two voxels may attribute to combination of two factors: different macro-dose in voxels and local radiation patterns, i.e. higher dose effect of low-energy electrons in the peripheral voxel than in the isocenter voxel. The proposed systematic model provides a scheme of taking local anatomical, micro-environmental and radiation information into consideration for a more “real” estimation of radiobiological effects, which is a step forward and advancement for modeling and translational researches as compared to the current macro-dose based LQ model.

**Figure 6:**
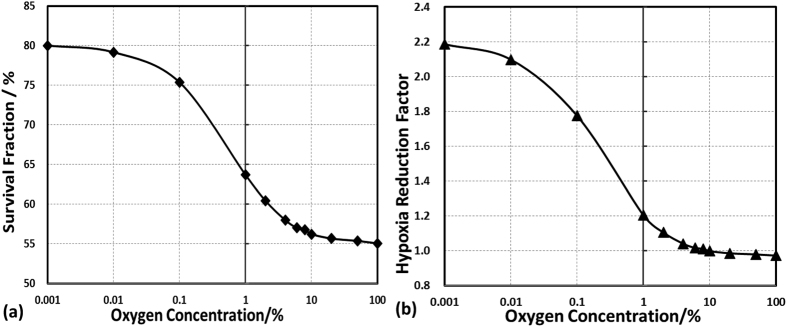
(**a**) The survival fraction and (**b**) hypoxia reduction factor (HRF) under various oxygen concentrations of the isocenter voxel after a single fraction irradiation.

For the isocenter voxel, SF under various oxygen concentrations was calculated and compared (Fig. 6(a)). SF increases significantly in reduced oxygen environment, especially in the concentration range from 10% to 0.1%. SF is calculated to be 80% in extreme anoxic condition (0.001%). As shown in Fig. 6(b), HRF increases monotonically and reaches a maximum value of 2.24 in extreme anoxic condition. The obtained HRF curve can be used as a quantitative reference to ensure the expected cell killings within the tumor under a certain oxygen microenvironment.

## Discussion and Conclusion

In this study, the radiation-induced cell killing effect was estimated with a new multi-scale model. Not only macroscopic deposited dose, but also individual patient anatomies, the radiation pattern at voxel level and the microscopic stochastic features of radiation were taken into account in the multi-scale biological effect estimation approach. Calculation of voxel-based cell killing effect for the chosen voxels has demonstrated the capability of the proposed model to connect radiation physics and underlying radiobiology. Currently, comprehensive *in vivo* measurement of “real” radiobiological effects cannot be achieved for patients. Modeling is the only way to help understand how the radiation interactions take place and quantify cellular damage caused by the deposited ionizing radiation energy. Compared with existing radiobiological estimation models (i.e. the widely used LQ model), the systematic model presented in this manuscript showed the same trend with considerable difference. It is reasonable to believe that the more radiobiological details are included in the systematic model, the closer to reality the estimated results are. Our mechanism-based systematic radiobiological estimation method provides a tool to bridge the physical quantity (i.e. dose expressed as energy deposited in tissue) and the “real” radiation-induced cell death in clinical radiotherapy treatment, which adds important value to development and advancement of personalized clinical radiotherapy techniques.

This study is motivated by the increasing evidence that RBE is greater than one for low energy electrons and photons^38^. For example, the RBE of characteristic ultrasoft X rays of carbon K (0.28 keV), copper L (0.96 keV), aluminum K (1.5 keV), and titanium K-shell (4.55 keV) are reported to be greater than 2 from radiobiological studies^20^,^39^. This suggests that the radiobiological effect may be strongly associated with higher dose-effect of low energy electrons, and energy spectrum plays a role in the radiobiological effects. The proposed multi-scale MC simulation approach aims to distinguish different radiobiological effects of specific treatment plan for individual patient, which is of great significance for personalized radiotherapy. To further investigate the influence of the energy spectrum on the radiobiological effect, six distinct spectrums of photon beams (nominal energy is 50 kV, 250 kV, 500 kV, 1 MV, 3 MV and 6 MV, respectively, extracted from^40^,^41^) were employed as incident source at voxel level. The macroscopic deposited dose was identical for all scenarios, i.e. 1 Gy. The microscopic damage patterns and cell survival fractions were calculated using the same procedures (as shown at Table 2). In the six irradiation situations, fractions of low energy electrons (<100 keV) were increased significantly with reduced nominal energy. The total DSB yields and fraction of complex DSBs were also different for various spectrums. Accordingly, final survival fraction decreases from 80.6% (6 MV x-rays) to 75.6% (50 kV x-rays). Compared to 6 MV x-rays, local relative biological effectiveness for 50 kV, 250 kV, 500 KV, 1 MV and 3 MV beams were calculated as 1.26, 1.12, 1.09, 1.06, 1.03, respectively. These results align with the understanding that lower energy X-rays could be more effective and cause more local cancer cell killings. A good example to reflect the increased radiobiological effect of low energy X-rays is gold nanoparticle enhanced radiotherapy (GNPT). The observed radiosensitizing effect comes from increased fraction of low energy electrons (photoelectrons and Auger electrons) at the vicinity of gold nanoparticles^42^,^43^. Therefore, this multi-scale modeling approach will be very useful for understanding the potential influence on biological effects of investigations involving energy spectrum alteration, such as low-Z target switching technique in the gold nanoparticle radiotherapy^44^ or the applications of lower MV photon beams in therapy or imaging fields^40^,^45^.

The radiobiological effects of oxygen concentration are investigated in this study. Molecular and cellular damages may become unrepairable and permanently damaged in the presence of oxygen. While under hypoxic condition, tumor cells may show some resistance to radiation, which is a leading factor towards radiotherapy treatment failure. The introduction of oxygen effect in the personalized treatment outcome estimation procedure may be necessary and of great significance. In addition, more tumor microenvironment information of the patients can be added in the cellular damage database from both *in vitro* and *in vivo* studies. Voxel-based functional images, such as the magnetic resonance imaging (MRI)^46^,^47^, positron emission tomography (PET), radiolabeled antibody imaging and the fusion images of multiple modalities, could be implemented into the multi-scale simulations in the future to provide more microenvironment information of 3D biological characterization. It is well recognized that the more specific biological information is implemented into the modeling, the more reliable results one can get.

The framework for biological effect estimation proposed in this study has the potential to be incorporated into the radiotherapy clinic if the radiation patterns could be extracted from other “fast” Monte Carlo systems in the future^48^. For individual patients, after general fast optimization procedure, the final dose distribution can be generated using MC simulations. Before the simulation, one or more ROIs, such as potential hypoxia area within the tumor or the “cold spots” in the plan, can be selected for specific treatment plan for the individual patient. As discussed in this work, during the MC simulation, the radiation pattern of the chosen ROIs can be saved and used for further biological effect estimation. The cell survival fraction of the ROIs can be calculated to evaluate plan quality and estimate possible treatment outcomes for the individual patient.

In conclusion, a systematic approach is developed and multi-scale Monte Carlo (MC) simulations from organ to cell levels are performed. Not only the macroscopic deposited dose, but also the individual patient anatomies, radiation pattern at voxel level and the microscopic stochastic features of radiation are accounted for in the radiobiological effect estimation procedure. The results of our feasibility study are in accordance with expectations based on radiobiological principles and reported studies.

## Additional Information

**How to cite this article:** Zhang, Y. *et al*. An Expanded Multi-scale Monte Carlo Simulation Method for Personalized Radiobiological Effect Estimation in Radiotherapy: a feasibility study. *Sci. Rep.* **7**, 45019; doi: 10.1038/srep45019 (2017).

**Publisher's note:** Springer Nature remains neutral with regard to jurisdictional claims in published maps and institutional affiliations.

## Figures and Tables

**Figure 1:**
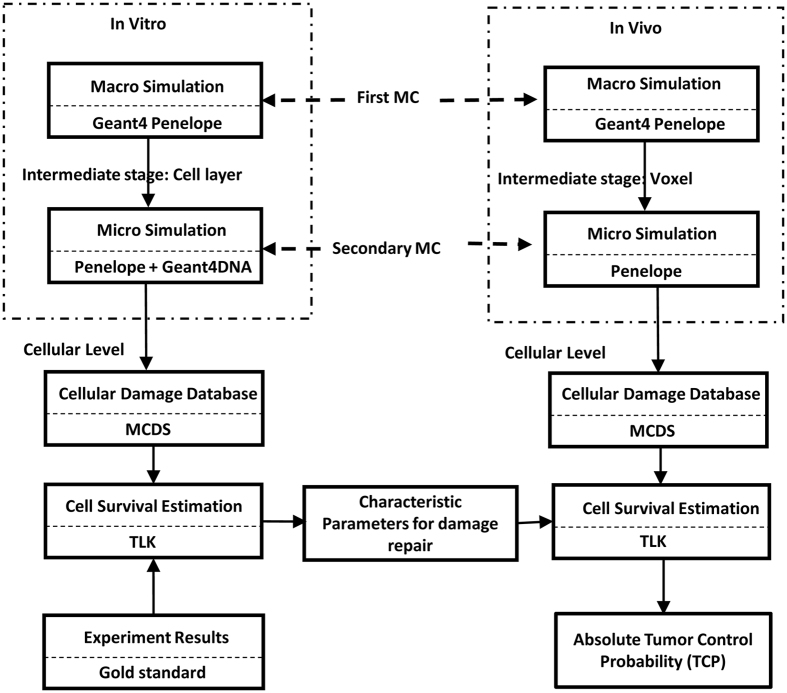
Flowchart of the expanded multi-scale Monte Carlo simulation.

**Table 1:** Radiation features of two chosen voxels under normoxic condition (21%).

	Isocenter Voxel	Peripheral Voxel
Macroscopic Features of the Voxel:		
Density (g/cm^3^)	1.07	0.28
Material	Soft tissue	Lungs inhale
Mean Energy of incident electron (keV)	656	594
Mean Energy of incident photon (keV)	1292	1197
Voxel Dose from TPS (Gy)	2.21	2.12
Voxel Dose from Geant4 (Gy)	2.2	2.06
Microdosimetric Features among all nucleus:		
Fraction of electrons <100 keV (%)	19.8 ± 3.9^c^	20.6 ± 4.2^c^
Nucleus Dose (Gy)	2.07 ± 0.50^c^	1.97 ± 0.52^c^
Yield of Total DSB	111.5 ± 24.1^c^	109.8 ± 25.3^c^
Yield of cDSB	59.6 ± 17.4^c^	60.4 ± 15.2^c^
Yield of sDSB	51.7 ± 10.1^c^	50.7 ± 10.4^c^
Survival Factor per Fraction:		
SF_LQ (%)^a^	51.7	54.3
SF_MC(%, 24 h)^b^	55.8 ± 0.5^d^	57.9 ± 0.4^d^

^a^Survival fraction calculated by LQ model using the macroscopic dose of the voxel.

^b^Survival fraction calculated in this study after 24 hours of damage repair.

^c^Mean ± standard deviation among a number of 10^5^ nucleus models. The values were averaged using results from three repeated simulations.

^d^Mean and standard deviation obtained from three repeated simulations.

**Table 2:** Radiation features of six spectra irradiations under normoxic condition (21%).

	50 kV	250 kV	500 kV	1 MV	3 MV	6 MV
Mean photon energy (kV)	30.50	123.80	184.80	375.10	897.50	1871.00
Fraction of electrons <100 keV (%)	100 ± 0.33	98.43 ± 1.69	78.12 ± 4.81	33.89 ± 4.68	15.93 ± 2.93	9.53 ± 1.79
Nucleus Dose (Gy)	0.93 ± 0.20	0.92 ± 0.14	0.9 ± 0.13	0.87 ± 0.11	0.85 ± 0.09	0.85 ± 0.09
Yield of Total DSB	52.54 ± 11.67	49.86 ± 7.64	48.02 ± 6.81	45.47 ± 5.94	44.95 ± 5.12	44.77 ± 4.85
Yield of cDSB	31.42 ± 5.00	28.61 ± 4.24	26.4 ± 3.79	24.64 ± 3.34	24.02 ± 2.95	23.33 ± 2.78
Yield of sDSB	23.12 ± 4.77	23.26 ± 3.44	21.62 ± 3.06	20.64 ± 2.64	20.77 ± 2.29	21.44 ± 2.11
Fraction of cDSB	0.60	0.57	0.55	0.54	0.53	0.52
SF_MC(%, 24 h)^a^	75.63 ± 0.71	78.27 ± 0.75	79.02 ± 0.74	79.48 ± 0.77	80.13 ± 0.75	80.66 ± 0.79
Relative Biological Effectiveness^b^	1.26	1.12	1.09	1.06	1.03	1.00

^a^The survival fraction calculated in this study after 24 hours of damage repair.

^b^RBE is cell survival fraction compared to results of 6MV photon beam. The values were averaged using results from three repeated simulations.
